# Whole-Genome Sequence of Limosilactobacillus fermentum Strain DM075, Isolated from the Human Oral Cavity

**DOI:** 10.1128/mra.00819-22

**Published:** 2022-10-31

**Authors:** Do-Young Park, Jiyoung Hwang, Yunji Kim, Young-Youn Kim, Hye-Sung Kim, Inseong Hwang

**Affiliations:** a DOCSmedi Co., Ltd., Goyang-si, South Korea; b Apple Tree Institute of Biomedical Science, Apple Tree Medical Foundation, Goyang-si, South Korea; University of Rochester School of Medicine and Dentistry

## Abstract

Here, we report the complete genome sequence of nitric oxide (NO)-producing Limosilactobacillus fermentum strain DM075, which was isolated from human tongue coating samples from healthy donors in South Korea. The complete genome sequence of DM075 comprises a single circular 2,204,022-bp genome, with a GC content of 51.0%, and lacks antimicrobial resistance genes.

## ANNOUNCEMENT

The relationship between oral microbiota and their production of nitric oxide (NO) has recently gained much attention (1–6). To isolate NO-producing *Lactobacillus* strains, we obtained tongue coating samples from healthy donors from South Korea, measured nitrate-reducing activity using a double agar overlay method based on the Griess reaction (4), and identified DM075 by complete 16S rRNA gene sequencing in February 2022.

For long-read sequencing, DM075 was anaerobically and statically cultivated in MRS broth for 24 h at 37°C. Genomic DNA (gDNA) was extracted using the Maxwell RSC system (Promega, USA) and sheared to 7 to 12 kb using the Megaruptor 3 system (Diagenode, USA), and small fragments (<3 kb) were removed using AMPure PB beads (Pacific Biosciences [PacBio], USA). The sequencing library was constructed using 3 μg of gDNA with the SMRTbell Express template preparation kit v2.0 (PacBio). The library was sequenced using the Sequel system (PacBio), yielding a total of 78,320 subreads (*N*_50_, 10,359 bp) with an average length of 8,323 bp. The data were assembled according to the Microbial Assembly protocol in SMRT Link v10.1.0.119588 (PacBio), including read quality control, error correction, adapter filtering, circularity checking, and overlap trimming (7), which produced a single circular *oriC*-rotated genome. For short-read sequencing, 100 ng of gDNA was sheared using Adaptive Focused Acoustics technology (Covaris, USA), and an ~350-bp sequencing library was prepared using the TruSeq Nano DNA high-throughput library preparation kit (Illumina, USA). The HiSeq X Ten platform (Illumina) was used for the sequencing, which resulted in a total of 17,586,382 quality-filtered 2 × 151-bp paired-end reads (with ≥90% of bases having Phred quality scores of >30). The Illumina reads were then mapped to the PacBio-assembled genome using BWA-MEM v0.7.17 (8) after adapter and quality trimming with Trimmomatic v0.38 (9). The final error correction was conducted three times with Pilon v1.21 (10) with a default minDepth value of 0.01, resulting in a single circular 2,204,022-bp chromosome (Table 1).

**TABLE 1:** Summary of assembly and annotation statistics for L. fermentum DM075

Parameter	Finding
Genetic element	Chromosome
Length (bp)	2,204,022
GC content (%)	51.0
No. of coding sequences	2,129
No. of rRNAs	15
No. of tRNAs	58
Sequencing depth (×)	259.2
GenBank accession no.	CP100352

The average nucleotide identity (ANI) was analyzed using OrthoANI (11), which yielded 99.28% sequence similarity with Limosilactobacillus fermentum CBA7106 (GenBank accession number CP021964.1). The genome annotation by the NCBI Prokaryotic Genome Annotation Pipeline (PGAP) v6.1 (12) predicted 2,129 protein-coding genes, 15 rRNA genes, and 58 tRNA genes. Additional annotations were conducted using Prokka v1.14.6 (13), InterProScan v5.30-69.0 (14), and eggNOG DB v4.5 (15), which suggested the highest incidence of genes for DNA metabolism (16.5%), followed by amino acid metabolism (8.6%) and translation (6.5%). The absence of antibiotic resistance genes, one of the key prerequisites for strain safety, was verified using ResFinder v4.1 (16) and the Comprehensive Antibiotic Resistance Database (CARD) v3.2.3 (17). All tools were run with default parameters unless otherwise specified.

The biospecimens used for this study were provided by the Biobank of Apple Tree Dental Hospital, a member of the Korea Biobank Network, after approval from the public institutional review board (http://public.irb.or.kr) (approval number P01-202111-31-002).

### Data availability.

The accession numbers for the 16S rRNA partial sequence and the genome sequence and raw sequencing reads for DM075 are as follows: GenBank, OP579185.1 and CP100352; BioProject, PRJNA853106; BioSample, SAMN29360653; SRA, SRX15901026 and SRX15901027.
